# Aggressive B-cell non-Hodgkin lymphomas: a report of the lymphoma workshop of the 20th meeting of the European Association for Haematopathology

**DOI:** 10.1007/s00428-023-03579-6

**Published:** 2023-08-02

**Authors:** Socorro Maria Rodriguez-Pinilla, Stefan Dojcinov, Snjezana Dotlic, Sarah E. Gibson, Sylvia Hartmann, Monika Klimkowska, Elena Sabattini, Thomas A. Tousseyn, Daphne de Jong, Eric. D. Hsi

**Affiliations:** 1 Pathology Department, Hospital Universitario Fundación Jiménez Díaz, Madrid, Spain; 2 Department of Pathology, Morriston Hospital, Swansea Bay University Health Board, Swansea, UK; 3 Department of Pathology and Cytology, University Hospital Centre Zagreb, Zagreb, Croatia; 4 Division of Hematopathology, Department of Laboratory Medicine and Pathology, Mayo Clinic, Phoenix, AZ USA; 5 Dr. Senckenberg Institute of Pathology, Goethe University Frankfurt Am Main, Frankfurt Am Main, Germany; 6 Department of Clinical Pathology and Cancer Diagnostics, Karolinska University Hospital, Stockholm, Sweden; 7 Haematopathology Unit, IRCCS Azienda Ospedaliero-Universitaria Di Bologna, Bologna, Italy; 8 Department of Imaging and Pathology, Translational Cell and Tissue Research Lab, KU Leuven, Leuven, Belgium; 9 Department of Pathology, Amsterdam UMC, Location VUMC, De Boelelaan 1117, 1081HV Amsterdam, The Netherlands; 10 Department of Pathology, Wake Forest University School of Medicine, Winston-Salem, NC USA

**Keywords:** High-grade B-cell lymphoma, Burkitt lymphoma, High-grade B-cell lymphoma/large B-cell lymphoma with 11q aberration, Large B-cell lymphoma with IRF4 rearrangement, Diffuse large B-cell lymphoma/high-grade B-cell lymphoma with MYC and BCL2 and/or BCL6 rearrangements

## Abstract

Aggressive B-cell non-Hodgkin lymphomas are a heterogeneous group of diseases and our concepts are evolving as we learn more about their clinical, pathologic, molecular genetic features. Session IV of the 2020 EAHP Workshop covered aggressive, predominantly high-grade B-cell lymphomas, many that were difficult to classify. In this manuscript, we summarize the features of the submitted cases and highlight differential diagnostic difficulties. We specifically review issues related to high-grade B-cell lymphomas (HGBCLs) with *MYC* and *BCL2* and/or *BCL6* rearrangements including TdT expression in these cases, HGBCL, not otherwise specified, large B-cell lymphomas with *IRF4* rearrangement, high-grade/large B-cell lymphomas with 11q aberration, Burkitt lymphoma, and pleomorphic mantle cell lymphoma. Since the workshop, the 5th edition of the WHO Classification for Haematolymphoid Tumours (WHO-HAEM5) and International Consensus Classification (ICC) 2022 were published. We endeavor to use the updated terminology.

**Supplementary information:**

The online version contains supplementary material available at 10.1007/s00428-023-03579-6.

## Introduction

The conceptual framework of the diffuse aggressive B-cell non-Hodgkin lymphomas has evolved over the past few decades due to our ability to study immunophenotypic and molecular genetic characteristics and to correlate them with clinical and pathologic features. This has allowed continuous refinement of clinical and biologic entities that advances the field and promises better treatments and outcomes. Although these lymphomas are all composed of intermediate to large cells with open or “blastic” chromatin, the constellation of immunophenotype, gene expression, genetic structural abnormalities, DNA copy number alterations, and mutational features adds complexity and sometimes supersedes morphology. If one overarching statement can be made, it is that while hematopathology still remains grounded in routine morphology, current diagnosis and classification require a relatively intense application of advanced testing to do justice to the current state of knowledge.

Session IV of the workshop addressed aggressive B-cell non-Hodgkin lymphomas. Sixty-seven cases were placed into this session. Review of these cases by the panel revealed seven groupings based on diagnosis and highlighted many unresolved issues, varying practices, and points requiring clarification that are a manifestation of our incomplete understanding of these lymphomas. These groupings included (1) high-grade B-cell lymphomas with *MYC* and *BCL2* and/or *BCL6* rearrangement (R); (2) terminal-deoxynucleotidyl-transferase (TdT)-positive B-cell leukemia/lymphoma with or without “double/triple hit” genetics, (3) high-grade B-cell lymphomas, not otherwise specified (nos); (4) large B-cell lymphomas with *IRF4*-R; (5) Burkitt lymphoma; (6) high-grade/large B-cell lymphoma with 11q aberrations (HG/LBCL-11q) (formerly Burkitt-like lymphomas with 11q abnormalities (BLL 11q)); and (7) *CCND1*-R lymphomas, usually pleomorphic/blastoid mantle cell lymphoma. Other large B-cell lymphomas of varying types, generally single examples, were also submitted but will not be commented upon further. Diffuse large B-cell lymphoma, not otherwise specified (DLBCL, nos), was not covered in this session. We will describe the cases and themes for each of these seven groups. At the time of the workshop, the revised 4th edition WHO Classification (WHO-HAEM4R) terminology was in use. Where relevant, notation is made of differences in terminology in the recently published WHO Classification 5th edition [1] and International Consensus Classification (ICC) 2022 [2]. Diagnoses of the panel for cases in this session are listed in the supplemental Table 1.

## High-grade B-cell lymphoma with MYC and BCL2 and/or BCL6 rearrangement

**Fig. 1:**
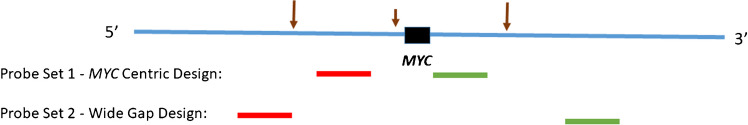
Schematic demonstrating *MYC* FISH break apart probe design. The black box represents the *MYC* locus on chromosome 8q14. Probe set 1, with a *MYC*-centric design narrowly flanks *MYC*. Probe set 2, with a “wide gap” design flanks *MYC* with larger region of intervening DNA. The short brown arrow represents a “genic” break point immediately upstream of *MYC* which typically involved *IGH* as the partner gene. It would be detected by splits on both probe sets 1 and 2. The long brown arrows represent “non-genic” breakpoints that are enriched for non *IGH* partner genes and would be detected by probe set 2 but not probe set 1. FISH, fluorescence in situ hybridization

High-grade B-cell lymphoma with *MYC* and *BCL2* and/or *BCL6*–R (DHL/THL) are defined by their genetic background and were recognized as highly aggressive B-cell lymphomas that can occur de novo or as transformations from prior lymphomas, most often FL or DLBCL, nos [3–7]. The histopathology is that of a diffuse lymphoma with variable cytology that can include conventional DLBCL, intermediate between DLBCL and Burkitt lymphoma (BL), and blastoid morphology [8]. While the proliferation is typically high (> 90%), this feature alone does not qualify a case for this category. The immunophenotype is typically that of a mature B-cell with a germinal center B-cell phenotype. Rearrangements in *MYC*, *BCL2*, and *BCL6* are identified by fluorescent in situ hybridization (FISH) but strategies to do this vary and may miss or misidentify some translocations [9–11]. Importantly, commercially available break apart (BA) FISH probes for *MYC* rearrangement detection differ significantly. Generally, *MYC* BA assays are used. The *MYC*-centric probes that target a smaller region centered on *MYC* will detect the so-called genic rearrangements with breakpoints located upstream of the *MYC* coding region and in intron 1 and enriched for *IGH* as the partner (approximately 80% of cases). However, it will miss “non-genic” breakpoints located mostly downstream of the *MYC* gene which may include *IGL* partners, rarely *IGH*, and *non-IG* partners such as *BCL6*, *ZCCHC7*, and *RFTN1.* Alternatively, a “wide gap” *MYC* probe design that flanks *MYC* by a large region will pick up most non-genic breakpoints as well (Fig. 1) [12]. Combining this with an *IGH::MYC* dual fusion will allow detection of additional cases of *MYC* rearrangement that have been reported but missed by BA strategy alone and would also have the added benefit of confirming *IGH* as the *MYC* partner [10]. Unsettled issues related to DHL/THL illustrated by submitted cases include phenotypic variation and characteristics of *MYC* and *BCL6* DHLs. The issue of a *MYC::BCL6* fusion resulting in a “pseudo”-DHL is also in need of clarification.

Twelve cases were submitted to the workshop and were split between DHL (7) (LYWS-258, 305, 657, 697, 734, 417, and 553) and THL (5) (LYWS-260, 453, 514, 685, and 721). Three of the DHLs were *MYC-* and *BCL6-*R (LYWS-258, 305, and 553) and four were *MYC-* and *BCL2*-R (657, 697, 734, and 417). One of the THL cases was, in fact, a *MYC*::*BCL6* “pseudo-”THL (LWYS-721). Ten were de novo and one each occurred in the background of follicular lymphoma (a THL) (case LYWS-260) and extranodal marginal zone lymphoma (DHL with *MYC* and *BCL6*-R) (case LYWS-305). Clonal identity between the low-grade and high-grade lymphomas was not proven in either case. The mean age of the patients was 60.5 years (median 63 years, range 34–89). The *BCL6* DHLs had DLBCL morphology and the *BCL2* DHL and THLs showed both DLBCL (4) and high-grade Burkitt-like or blastoid morphology (5). Immunophenotypically all but one case expressed CD10 and 8/9 tested cases expressed BCL6. One case, a *BCL6* DHL, was CD10 negative and expressed MUM1, concordant with the propensity of *MYC/BCL6* DHLs to be CD10-/MUM1 + compared to *MYC/BCL2* DHLs [13].

Features of two cases were problematic for the panel. First, case LYWS-258 (Garamvölgyi E, et al.; University Hospital, Basel, Switzerland) had an unusual immunophenotype. This was a de novo case in an 89-year-old man with cutaneous and retroperitoneal masses. This case had DLBCL, nos morphology and expressed CD5, CD10, BCL6, MUM1, MYC (90%), Ki67 (100%), and SOX11 but lacked cyclin D1. FISH showed *MYC* and *BCL6*–R without *BCL2* or *CCND1*-R. The panel felt this case was best considered a *MYC/BLC6* DHL with an unusual phenotype in WHO-HAEM4R. SOX11 is expressed in cyclin D1-negative MCL (classic and blastoid variant) but can be seen in other blastoid neoplasms such as Burkitt lymphoma [14]. Additionally, some SOX11 antibodies lack specificity in immunohistochemistry, and the primary antibody used in case 258 was not specified [15]. Studies for *CCND2* and *CCND3* expression may be of use to further exclude a cyclin D1-negative blastoid MCL [16].

Case LYWS-721 (Dojcinov S, et al. Department of Cellular Pathology, Cardiff and Birmingham University, UK) demonstrates an important pitfall and limitation of FISH testing for DHL/THL. The 74-year-old patient had a large axillary lymph node involved by a diffuse aggressive large B-cell lymphoma with a germinal center B-cell immunophenotype, high Ki67 index (80%) and expressed MYC (40%) and BCL2. FISH showed *MYC*, *BCL2*, and *BCL6* rearrangements by break apart probes. However, *MYC::BCL6* dual color dual fusion probes done as part of a comprehensive study from the submitters showed the presence of *MYC::BCL6* rearrangement. Such cases, with a t(3;8)(q27;q24), have been reported previously and represent “pseudo-double hit lymphomas.” This translocation may not be equivalent to conventional *MYC* and *BCL6* DHL/THL, and further characterization of the clinical and biologic features of such cases is required [9].

This case raises the larger question of what an appropriate FISH strategy might be for routine detection of DHL/THL. Additionally, an unresolved issue is whether the partner genes for *MYC* should be identified. *MYC* copy number does appear to confer the biology of translocation in the context of DHL [17]. A recent multicenter retrospective study with 264 cases of de novo diffuse large B-cell morphology treated with immunochemotherapy tested cases with a “wide gap” *MYC* BA probe, *BCL2* and *BCL6* BA probes, and commercially and non-commercially available *IGH::MYC* and *IGL::MYC* dual fusion probes. This study found that a *MYC-*R *with an IG* partner was associated with poor progression-free and overall survival [18]. Various other smaller and more heterogeneous studies have reported variable associations to outcome and further validations are awaited [18–23].

The concept of a molecular high-grade gene signature should be touched upon. Independently, two groups identified a gene expression profile of BL, noting that while cases of BL had this “molecular BL” (mBL) signature, a few DLBCL cases had this signature. Further, an intermediate probability group was also identified. These mBL signature DLBCLs and intermediate cases were enriched for cases with *MYC* and *BCL2* rearrangements [24, 25]. Later, molecular high-grade (MHG) or DHL signatures were described that were related to BL and some germinal center B-cell lymphomas with DLBCL morphology and poor prognosis. Such signatures recognize intermediate-to-dark zone centroblastic cells as opposed to light zone/centrocytes. Thus, these molecular high-grade signatures appear to recognize germinal center dark zone (DZ) biology and are highly enriched in DHLs, although a large fraction of such MHG/DHL signature cases lack *MYC* and *BCL2* rearrangements [26–28]. Whether application of molecular high-grade/mBL signatures can be applied routinely will require further investigation and enabling technologies to become more widely available.

Workshop cases highlight variability in the pathologic features of DHL/THL. FISH strategies should be carefully considered by laboratories with the realization that false negatives are more likely with use of *MYC* break apart probes that tightly flank *MYC*. While *MYC/BCL2* DHLs typically have a GCB immunophenotype, *MYC/BCL6* DHLs appear to more likely be CD10-/MUM1 + . CD5 expression is possible but mantle cell lymphoma (including *CCND1*-negative MCL) should be excluded. Furthermore, whether *MYC/BCL6* DHLs are distinct from *MYC/BCL2* DHLs and DLBCL, nos, is not settled. Finally, *MYC::BCL6* “pseudo”-DHLs deserve further study. Currently, pathologists and classification systems do not require identification of *BCL2*, *BCL6*, or *MYC* partner genes.

## TdT-positive B-cell leukemia/lymphoma with or without “double/triple hit” genetics

The revised WHO 4th edition recommends TdT + DHL/THLs be diagnosed as B-LBL. In this section, we review submitted TdT + cases with DHL/THL genetics and consider current thinking on this issue as well as the features of rare *MYC*-R B-LBL/leukemia. Sixteen cases (LYWS-296, 634, 699, 175, 474, 690, 738, 788, 245, 578, 622, 383, 225, 290, 234, and 268) of aggressive B-cell leukemia/lymphoma with TdT expression were submitted. By far, the largest group (13 cases) was (DHL/THL). Two cases of B-lymphoblastic lymphoma (B-LBL) with *MYC* rearrangement were submitted. The remaining case was a B-lymphoblastic leukemia and will not be discussed further. TdT is typically a marker of immaturity, being expressed in most precursor B- and T-cell lymphoblastic leukemias/lymphomas and uncommonly in myeloid blasts. It has been known for many years that TdT expression can be seen in unusual cases of DHL/THL, which could occur as a transformation of a prior low-grade lymphoma such as follicular lymphoma or as de novo disease [29–36]. Such cases have been reported to express CD19 and CD10 but often lack CD34 and CD20. They may or may not express surface immunoglobulin [30, 37, 38].

**Fig. 2:**
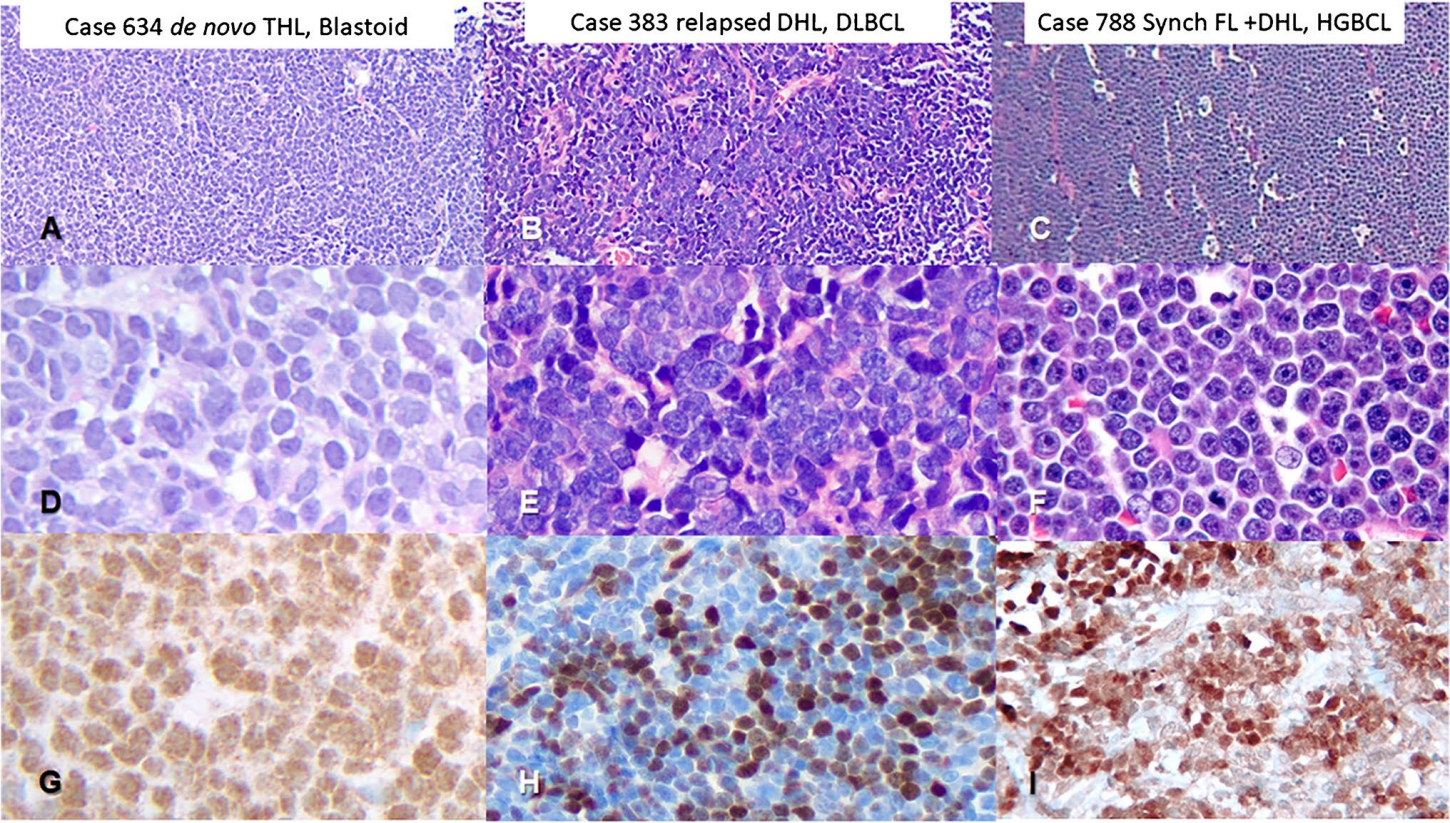
DHL/THLs with TdT expression. Low-magnification H&E images (**A**, **B**, **C**, 10 ×), high-magnification H&E images (**D**, **E**, **F**, 40 ×), and TdT immunostain (**G**, **H**, **I**, 40 ×) of double or triple hit lymphoma with TdT expression. These cases represent the morphologic spectrum of such cases including blastoid (**D**), diffuse large B-cell lymphoma (**E**), or high-grade B-cell lymphoma morphology (**F**)

In these 13 TdT + DHL/THL cases, the mean age was 62 years with a M:F ratio of 6.5. Three were de novo and ten had concurrent or history of a TdT-negative B-cell lymphoma. Seven of these ten patients had synchronous (3 patients) or prior follicular lymphoma (FL), one of which was a FL 3B. Two had relapse of a DHL that became TdT + . The remaining case without FL had a long history of CLL prior to the TdT + THL as a Richter transformation (LYWS-245, Wang, W. MD Anderson Cancer Center, Houston, TX). The TdT + lymphomas involved both nodal sites as well as extranodal sites such as bone marrow (1), upper aerodigestive tract (1), femur (1), chest wall (1), and CNS (2). Morphologically, eight cases were blastoid, three had diffuse large B-cell lymphoma nos features, and two had high-grade features (intermediately sized with small centroblastic features and starry sky appearance, Fig. 2). CD20 was expressed in all cases, CD10 was expressed in 12/13 cases, and CD34 was uniformly negative. TdT was expressed in 20–100% of cells (median 40%, mean 33%) with a range of intensity which was often variable within a case. However, three cases showed moderate to intense staining in 100% of cells. Nine cases were assessed for surface immunoglobulin (sIg) expression by flow cytometry. Six cases lacked detectable sIg while two cases expressed monotypic kappa and one case monotypic lambda. Genetically four were THL and nine were DHL (*MYC/BCL2* in eight and *MYC/BCL6* in one).

One case had mutational analysis (case LYWS-578, Bhavsar S, University of Pittsburgh, Pittsburgh, PA) and we applied a NGS customized panel (Sophia Genetics) in 6 cases (LYWS-788, 578, 290, 175, 738, and 234 cases) (Supplementary Methods, Supplemental Tables 2 and 3). For case 578, which was sequenced by the contributors and the panel, pathogenic *EZH2* and *TP53* mutations were found in both tests despite use of different assays with different gene coverage. *EZH2* mutation was also found in an additional case. *KMT2D* mutations were also seen in 2 cases (one with two mutations). The former is present in a substantial proportion of FLs but is not considered a driver mutation in B-lymphoblastic leukemia [39, 40]. Interestingly a *MYD88 L265P* mutation was present in LWYS-788 (Insuasti-Beltran G, Wake Forest University Medical School, Winston-Salem, NC). Again, this is not among recognized recurrent driver mutations in B-lymphoblastic leukemia. These data support the hypothesis that at least some of these cases may be related to a preexisting mature B-cell lymphoma background. As expected, therapy was heterogeneous. Of the cases with treatment data, five received HGBCL therapy and two ALL therapy. As a group, these were very aggressive neoplasms, with a median time to progression of only 4 months.

The proper terminology for such cases is debatable. The WHO-HAEM4R recommended these cases be diagnosed as B-lymphoblastic lymphoma [41]. This is problematic since, as illustrated by LYWS-175 (Insuasti-Beltran, Wake Forest University School of Medicine, Winston-Salem, NC) and 578 with a *MYC* and *BCL2* rearrangement, the genetic background resembles FL rather than LBL. Indeed, in a reported series of 6 cases of TdT + DHLs (*BCL2-*R in five and *BCL6*-R in one, combined with *MYC*-R), panel mutation testing showed mutation profiles more akin to germinal center B-cell derived DLBCL as opposed to B-LBL. With the caveat of a small sample set, mutations in *ARID1A*, *CREBP*, and *MEF2B* were seen in more than one case in that series. One case also had mutations in *EZH2* and *TNFRSF14*, common recurrent mutations in FL [37]. Detailed genetic analysis of transformed lymphomas DHLs with TdT expression supports this [42]. Interestingly, case LYWS-788 showed a *MYD88* mutation in the THL TdT + sample but suffered from a concomitant FL, a feature previously described in FL transformation [43]. Moreover, LYWS cases 738 (Llamas Gutierrez F, CHU de Rennes, Rennes, France) and 234 (Lee WS, University of Pennsylvania, Philadelphia, PA) showed *CCND3* and *ID3* mutations respectively. While associated with BL, they are not specific for BL since *CCND3* and *ID3* mutations have been reported in DHLs. These cases were not considered BL but rather DHL (*MYC-*R/*BCL2*-R) with TdT expression in a 75-year-old man and an unusual *MYC*-R B-LBL (*BCL2* and *BCL6* non-rearranged, see below) in a 69-year-old man, respectively [44, 45]. Thus, these DHL/THL cases are biologically different from de novo B-LBL. The panel felt that new terminology is required for these cases similar to that suggested by Ok and colleagues to distinguish them from B-LBL and denote the presence of DH or TH rearrangements [38]. Designating these cases as DHL/THL and qualifying with TdT expression (e.g., “High grade B-cell lymphoma with *MYC* and *BCL2* rearrangements, and expression of TdT”) is preferred by the panel [46]. Whether the standard workup of a DHL/THL would require TdT evaluation is also debatable. Given that this is not the current standard practice, it is likely some cases are missed in routine practice and the panel did not feel there was sufficient evidence to recommend routine assessment in DHL/THL. However, testing for TdT would help identify cases for further study.

True *MYC-*R precursor B-cell lymphoblastic leukemia/lymphomas do exist. Recent studies have begun to characterize these cases and show them to be distinct from BL, with *MYC-*R being the sole defining cytogenetic abnormality and association with *KRAS* mutations [47, 48]. One case of B-lymphoblastic lymphoma with *MYC*-R without *BCL2* or *BCL6*-R was submitted (LYWS-290, Lorsbach and colleagues, Cincinnati Children’s Hospital). It presented in the abdomen of a 10-year-old boy. Neoplastic cells expressed TdT and CD10 but lacked CD20 and CD34 by flow cytometry. Moreover, it expressed monotypic surface lambda and had a Burkitt lymphoma morphology. It contained an *IGH::MYC* rearrangement and NGS sequencing identified a *NRAS* Q61K mutation, confirmed by the panel. Case 234 was a TdT + blastic lymphoma occurring in a 69-year-old man that harbored a *MYC*-R and had a phenotype compatible with blasts (CD45 dim, CD10 + , PAX5 + , CD20 dim/neg, CD99 + , surface immunoglobulin negative) that also was felt to represent a B-LBL. *MYC*-R B-LBL/leukemia is extremely uncommon. In a series review from Pediatric Oncology Group, five of 5280 acute lymphoblastic leukemia cases were identified with *MYC*-R and precursor B-cell phenotype (0.09%). Patients responded ultimately to B-cell (Burkitt-type) therapy [49]. Interestingly, detailed molecular and epigenetic studies in 12 cases of Burkitt leukemia/lymphoma with an immature immunophenotype provide evidence that such cases resemble acute lymphoblastic leukemia (ALL)/lymphoblastic lymphoma (LBL) rather than BL. These pediatric cases appear to have *IGH::MYC* rearrangements resulting from aberrant *VDJ* recombination compatible with the rearrangement occurring in a precursor B-cell rather than a germinal center B-cell undergoing class switch or somatic hypermutation as one sees in BL. Furthermore, epigenetic analysis showed these cases clustered with ALL rather than BL cases [48]. Interestingly, either *BCL2* and/or *BCL6* gene rearrangements are present in a minority of cases [50].

In summary, this section highlighted issues around DHL/THL with TdT expression. Emerging data, supported by sequencing of a few cases from the workshop, argue *against* considering these cases as B-lymphoblastic lymphoma. The submitted cases frequently have a concurrent or a prior history of FL. TdT expression can be variable, and these cases express typically CD10 and CD20 but lack CD34. Considering them as DHL/THLs with the qualifier that they express TdT seems appropriate, rather diagnosing these cases as B-LBL. Further study of such cases is warranted. The rare occurrence of B-LBL/leukemia as part of the spectrum of TdT + blastic neoplasms that harbor *MYC-*R was illustrated*.*

## High-grade B-cell lymphoma, NOS

**Fig. 3:**
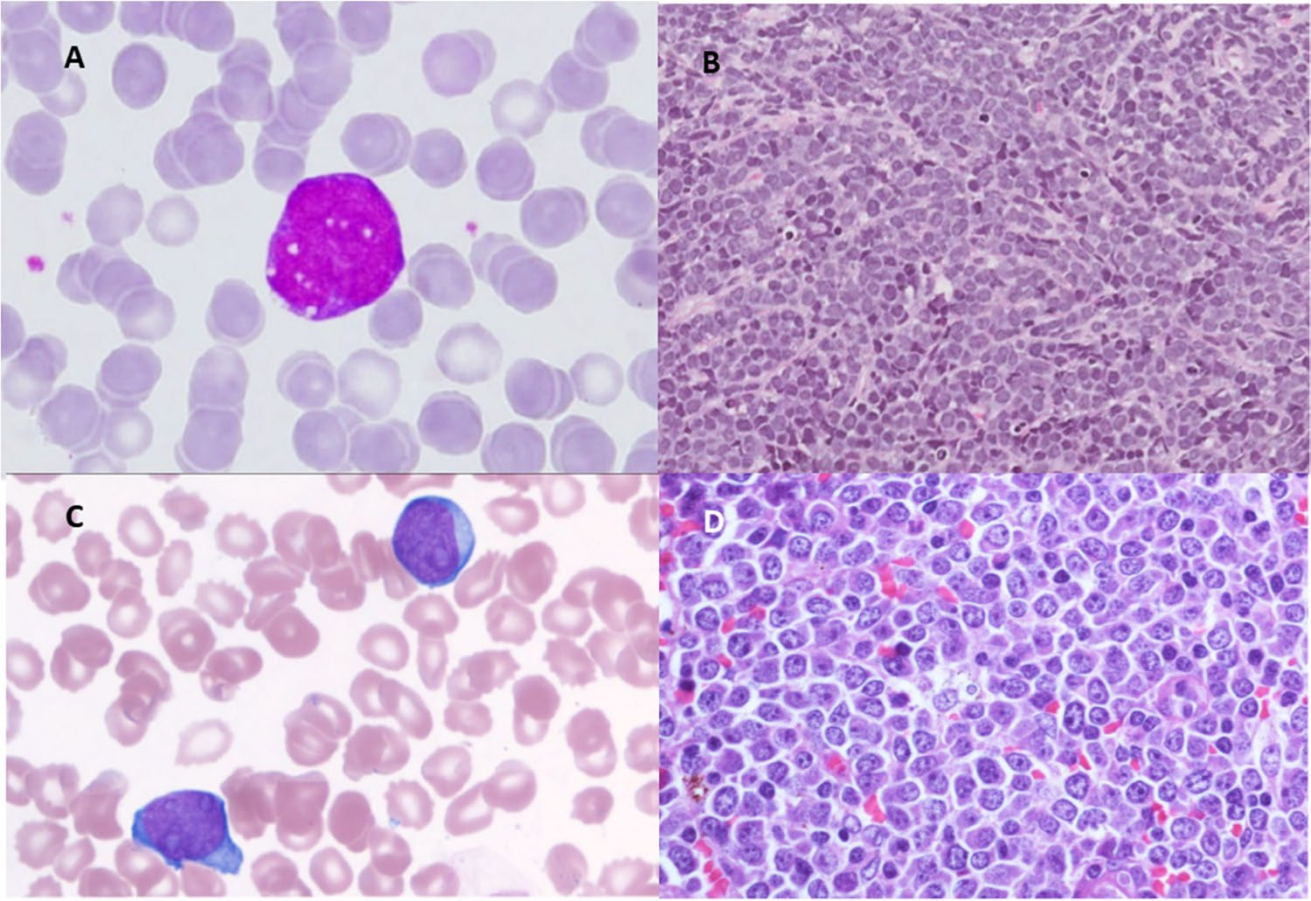
High-grade B-cell lymphoma, nos, case 759: **A** Peripheral blood with leukemic cells (100 ×). **B** Pelvic mass with blastoid morphology (H&E 40 ×). This patient was a 55-year-old woman who presented with a pelvic mass and bone marrow involvement. Flow cytometry showed a bright CD45 + B-cell population (CD19 + , CD10 + . CD20-, CD22 + , CD34-, TdT-, BCL2-, surface immunoglobulin negative). *IGH::MYC* rearrangement was present by FISH and *t(8;14)* was seen by karyotyping. Case 794: **C** Peripheral blood involvement (100 ×). **D** Cervical lymph node biopsy showing high-grade, Burkitt-like morphology (H&E, 40 ×) with a tingible body macrophage (center). This patient was 62-year-old woman who presented with splenomegaly and generalized lymphadenopathy. The cells express CD20, PAX5, BCL2, and MUM1 with near 100% MYC staining. The cells were negative for CD5, CD10, and BCL6. *IGH::MYC* but no *IGH::BCL2* or *BCL6*-rearrangement was seen

Eight cases were submitted to the workshop as high-grade B-cell lymphoma (HGBCL), nos according to WHO-HAEM4R criteria (LYWS cases 387, 440, 759, 778, 794, 532, 595, 363) [8]. HGBCL, nos is recognized as an imprecise and heterogenous category with somewhat subjective morphologic “high grade” features as the main defining characteristic at the present time. It is a diffuse lymphoma, typically occurring in older adults, with either blastoid and/or intermediately sized cells resembling those seen in BL (small centroblastic cells) (Fig. 3). A “starry sky” background is typically present. Issues illustrated in this section include the need to adhere to strict morphologic criteria and appropriate workup of cases, realizing that HGBCL, nos are considered a diagnosis of exclusion.

Of note, pediatric diffuse aggressive lymphomas can have some intermediate features and in-depth characterization is of utmost importance to exclude BL or DLBCL and cases should preferably not be classified as HGBCL, nos [8, 51]. Two of the eight cases submitted (LYWS-532, Uner A, et al. Duzen Laboratories, Ankara, Turkey; and LYWS-363, Wilson CS, et al. University of New Mexico Health Sciences Center, Albuquerque, USA) were pediatric cases, both with morphology that was felt to be more in keeping with DLBCL, nos. The third pediatric case (LYWS-595, Shafernak K et al. Phoenix Children’s Hospital; Phoenix, AZ, USA) was felt to represent a primary DLBCL of the central nervous system (CNS), which are extremely uncommon, and virtually only reported as case reports [52–55].

The remaining 5 cases (LYWS-387, 440, 759, 778, and 794) were from adults with a median age of 62 years, from three males and two females. All cases had either a “Burkitt-like” cytology resembling small centroblasts but with more variation than BL or blastoid cytology, and all had a starry sky pattern. One of the males (LYWS-440 case. Liu F. et al. Foshan Hospital, Sun Yat-sen University, Foshan, China) was HIV + and young at presentation (33 years). Viral load was not mentioned at presentation and the lymphoma was EBV-negative. This case had blastoid morphology, expressed CD10 but lacked BCL2 and was negative for *MYC*-R. Notably, the case lacked strong MYC expression (10%) but had a high Ki67 index (95%). Investigation for 11q abnormalities was not done and the workup of the case was considered incomplete. However, if it did not showed 11q abnormalities, it might be considered in the spectrum of HGBCL, nos. The remaining four could be considered by the panel as bona fide HGBCL, nos. Two had blastoid morphology and two had intermediate or Burkitt-like morphology and a starry sky pattern was present in all four cases. All were tested for DHL/THL genetics and three did harbor an isolated *MYC-*R. Three of four were germinal center B-cell phenotype according to the Hans algorithm. The one non-GCB case did harbor a *MYC*-R and lacked both CD10 and BCL6, while expressing MUM1. One case had cytogenetic analysis that revealed a complex karyotype including a t(8;14). MYC was expressed in 70% or more of the cells in all cases and two strongly expressed BCL2. The Ki67 index was 80–100% in 3 cases and 60% in the one non-GCB case. Thus, one can see some heterogeneity to these cases, as expected.

How, then, does one appropriately identify these cases? Submitted cases and our experience suggest that one must have strict morphologic criteria. From a practical standpoint, HGBCL, nos are a diagnosis of exclusion in which a lymphoma with intermediate or blastoid cytomorphology and starry sky pattern triggers a “high grade” workup to exclude BL, HG/LBCL-11q, and HGBCL with *MYC-* and *BCL2-* and/or *BCL6*-rearrangement. Thus, molecular studies (usually FISH) to exclude DHL/THL are mandatory. Also, FISH or other methods to exclude the 11q aberration should also be considered, particularly in cases of a background of tingible body macrophages and course apoptotic debris [56]. Cases with a starry sky but inappropriate cytology such as large centroblasts, and immunoblasts are not part of the HGBCL, nos spectrum. We should be reminded that the purpose of this entity is to serve as a placeholder for those cases that are currently insufficiently molecularly characterized to be separated into lymphoma entities but are felt to more likely have an aggressive course and poor prognosis to standard therapies. It is noteworthy that mBL/DHIT gene expression signatures appear to also recognize many, if not most, non-DH/THL with high-grade morphology [25, 27]. Conceptually, these signatures seem to identify dark zone germinal center B-cell expression patterns, and this may be a unifying theme to high-grade B-cell lymphomas, which may have varied morphologic features.

## Large B-cell lymphoma with IRF4 rearrangement

**Fig. 4:**
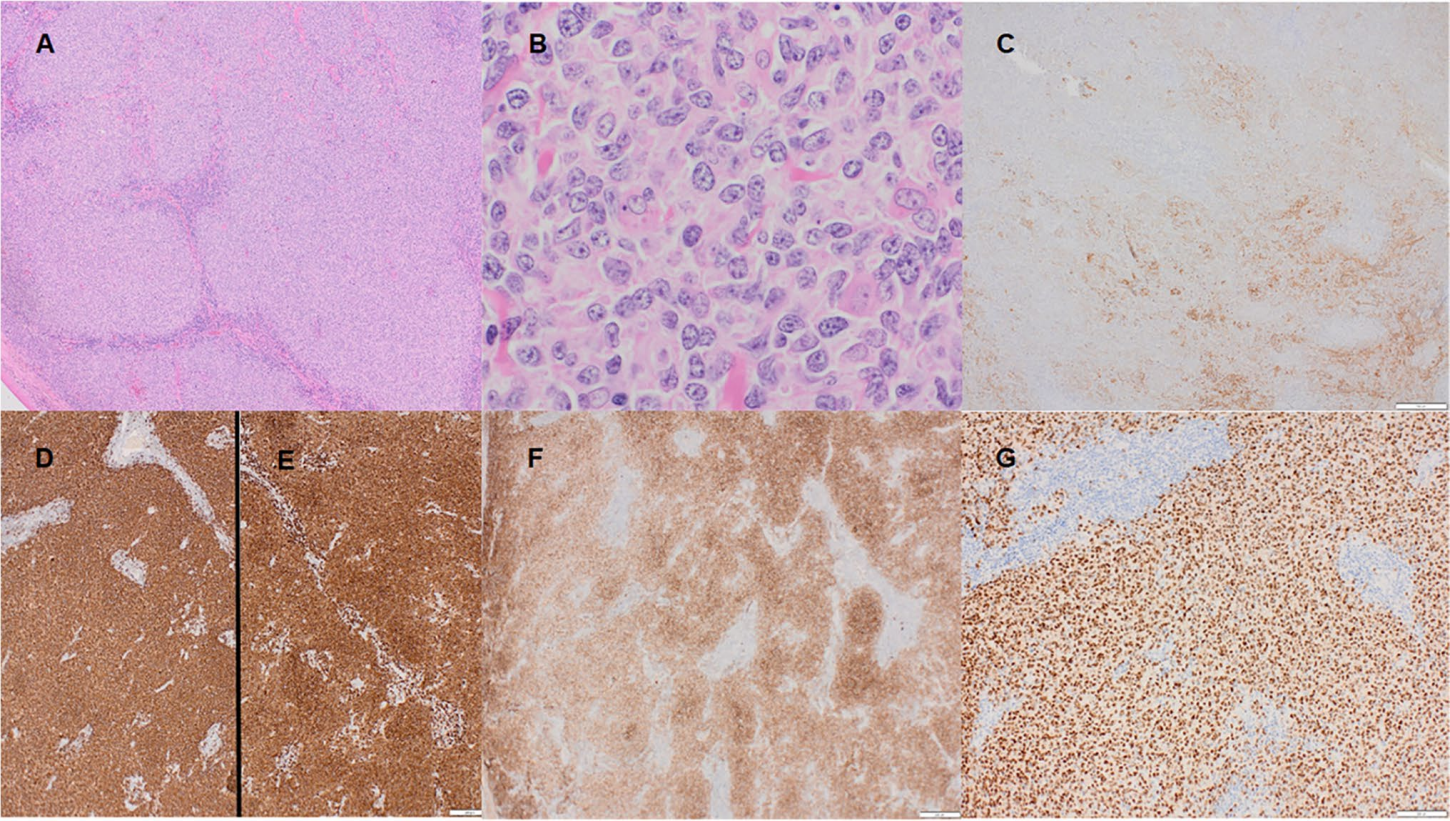
Large B-cell lymphoma with *IRF4*-rearrangement. **A** H&E, 4 × ; **B** H&E, 60 × , **C** CD21 immunostain, 4 × ; **D** CD20 immunostain, 4 × ; **E** CD5 immunostain, 4 × ; **F** CD10 immunostain, 4 × ; **G** MUM1 immunostain, 20 × . This case expressed BCL6 as well (not shown)

Large B-cell lymphoma with *IRF4* rearrangement occurs most commonly in children and young adults, often in the tonsil or head and neck region. They have a large B-cell/centroblastic cytomorphology and may either have a diffuse or follicular growth pattern. Recent studies note that cases may also be found in the adult population [57]. Immunophenotypically, large B-cell lymphomas with *IRF4* rearrangement express BCL6 and strong MUM1/IRF4 with the majority, but not all, also expressing CD10 (Fig. 4). CD5 may occasionally be seen and BCL2 is usually present [58, 59]. Patients generally have a favorable prognosis [58, 59]. Recent molecular genetic studies have demonstrated frequent mutations in *IRF4* and NFκB pathway genes (*CARD11*, *CD79B*, and *MYD88*), losses of *17p13*, and gains of chromosome 7, 11q12.3-q25 [60]*.* Adult cases may have more genetic complexity [57].

Five cases were submitted to the workshop, with four (LYWS cases 177, 190, 223, and 506) nicely fitting the described features of young age (≤ 30 years), diffuse architecture, and large to intermediately sized cells coexpressing BCL6 and *strong* MUM1, with three expressing CD10 and three expressing BCL2. CD5 was expressed in one case. *IRF4* was rearranged by FISH testing. One unusual case (LYWS-378, Quintanilla-Fend L, et al.; Institute of Pathology, Universitätsklinikum; Túbingen) was from a 70-year-old man with a mediastinal mass. This case expressed CD10, BCL6, and MUM1 but lacked CD5 and BCL2. However, in addition to an *IRF4*-R, a *CCND1*-R was present by FISH and cyclin D1 was expressed by immunohistochemistry. The patient was treated with multiagent immunochemotherapy but progressed at 5 months. Interestingly, the mutational analysis revealed *CARD11* and *IRF4* mutations are often seen in LBCL with *IRF4*-R [60]. Given the unusual clinical picture (elderly patient with a mediastinal mass), whether this case represents a LBCL with *IRF4*-R is uncertain but was favored by the panel [57]. Secondary *CCND1-*R have been reported to rarely occur, and might also explain the aggressive clinical course [61].

Given their favorable prognosis, recognition of these cases is important at least in the younger population. How should pathologists identify large B-cell lymphomas with *IRF4-*R? It is recommended that all DLBCL and FL 3B cases seen in the pediatric, adolescent, and young adult population (< 40 yrs) that coexpress BCL6 and MUM1 be screened for *IRF4-*R as is supported by a recent study [57].

## Burkitt lymphoma (BL)

**Fig. 5:**
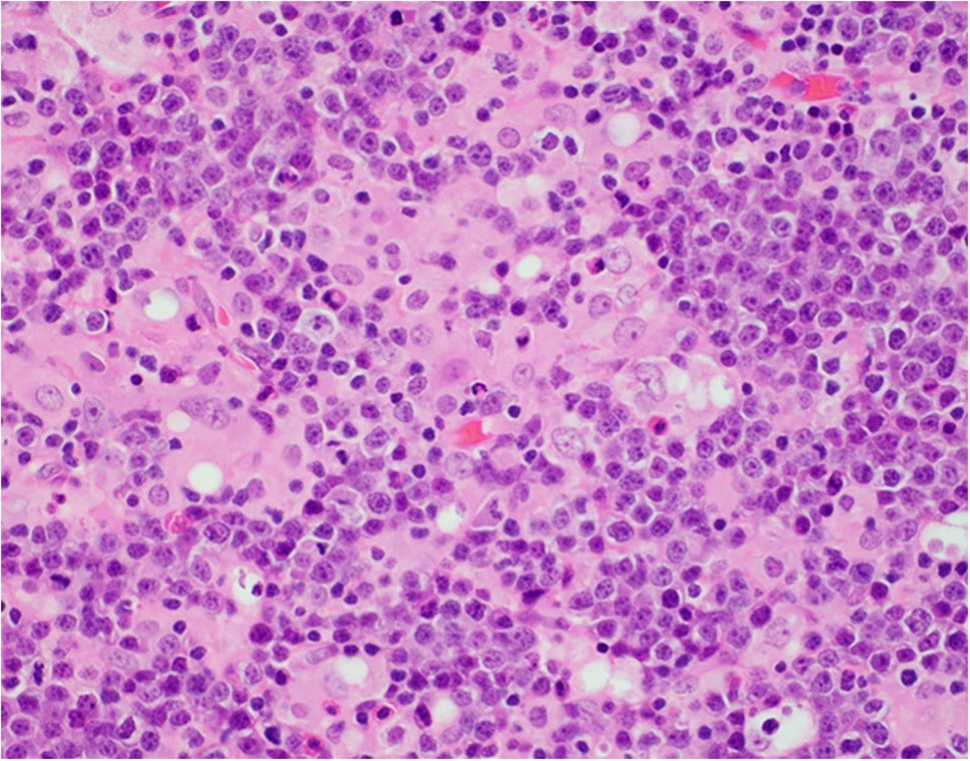
Burkitt lymphoma with granulomatous reaction, case 184. H&E 40 × , typical small centroblastic cells of Burkitt lymphoma with admixed granulomas

BL is characterized by a monotonous proliferation of small centroblasts with a background starry sky pattern. The characteristic immunophenotype is a mature B-cell expressing CD10, CD19, CD20, BCL6, and surface immunoglobulin that lacks BCL2 protein and shows a near 100% proliferative fraction [41]. EBV is seen in virtually all endemic cases while it is present in 20–30% of cases. Submitted cases showed peculiar but instructive clinical, morphological, or immunohistochemical features, reflecting “workshop bias.” We received three cases submitted as adult BL cases (LYWS-184, 331, and 510). There were two males and one female aged 51, 44, and 56 years old respectively. None of the patients suffered from known immunodeficiency. LYWS-184 case (Huang Q. Pathology Department; Cedars-Sinai Medical Center; Los Angeles, USA) showed unusual clinical and histologic features. It was from a 51-year-old man who suffered from waxing and waning systemic lymphadenopathy for more than 8 years. Morphologically, a granulomatous effacement of the lymph node architecture was found, with monomorphic medium-sized blastic cells in the background. These cells showed small amounts of basophilic cytoplasm and a high proliferation index (Fig. 5). Such cases of BL with a granulomatous inflammatory reaction have been reported in the literature as a rare variant of BL, should be differentiated from tuberculous lymphadenitis, are related to EBV type I latency, and are associated with a favorable prognosis and occasional spontaneous regression [62–64].

LYWS-331 case (Climent F, et al.; Pathology Department; Hospital Universitari de Bellvitge-IDIBELL; Barcelona; Spain) involved the jaw, breast, skin, and bone. The neoplastic cells showed BCL2 immunoreactivity. BCL2 is classically negative in BL cases; nevertheless, some series describe up to 23% of cases to express BCL2, although expression varied both in intensity and number of positive cells. No differences regarding either clinical or molecular features have been found between BCL2-positive and BCL2-negative BL cases [65]. The BL diagnosis in this case was supported by detection of *ID3* and *TP53*, both common (seen in approximately 40% of cases) in BL, with the former characteristic of BL [66]. In LYWS-510 (Fontaine J, et al. Pathology Department; Hospices Civils de Lyon, Pierre Bénite; France), the neoplastic cells had BL morphology, expressed CD10 and lacked BCL2, showed *MYC-*R, but had no MYC immunoreactivity. Rare BL cases lacking MYC protein expression despite the presence of a *MYC-*R have been reported. Two possible mechanisms for this have been described. One involves *MYCN* mRNA and protein expression, suggesting a switch to *MYCN*. The second involves lack of both MYC and MYCN proteins but shows *MYC* mRNA with mutations in *MYC* affecting the binding site of the MYC immunohistochemical primary antibody. These two groups showed overlapping clinical, morphologic, and immunohistochemical characteristics [67]. Interestingly, further FISH studies on LYWS-510 showed, in addition to *MYC* translocation, the 11q aberration (gain of 11q23.3 and loss of 11q24.3). The aberration was confirmed by chromosomal microarray studies. Classification of such rare cases is controversial, and the few studied cases suggest a common finding of 1q gains and lower genetic complexity compared to cases with 11q aberration but without *MYC*-R. Thus, these may be more closely related to BL and perhaps best diagnosed as BL [56, 68–70].

The differential diagnosis of BL includes HGBCL/DLBCL with *MYC* and *BCL2* gene rearrangements (either de novo or as a transformation of a follicular lymphoma), B-lymphoblastic leukemia/lymphoma and high-grade/large B-cell lymphoma with 11q aberrations. The typical phenotype of BL (CD19 + /CD10 + /BCL2-/Ki67 > 95%/TdT-/CD34-/sIg +) with appropriate cytomorphology, demonstration of *MYC*-R alone, and simple karyotype differentiates *MYC/BCL2* “double hit” lymphomas and lymphoblastic leukemia/lymphoma [50]. The differential diagnosis with high-grade/large B-cell lymphoma with 11q aberrations should be considered in cases lacking *MYC-*R and is further discussed below. The submitted cases emphasize two points. Variable, generally weak, BCL2 expression in BL, as shown in case 331, is allowable in BL but genetic support with *MYC* rearrangement involving an immunoglobulin gene partner should be present. Supporting mutational data, while not required, would further solidify the diagnosis. Finally, lack of MYC protein by IHC in the face of a known *MYC*-R and presence of morphologic and immunophenotypic features characteristic of BL should not dissuade one from diagnosing BL. Further study of mechanisms such as mutation of the epitope targeted by the primary antibody for IHC or switch to *MYCN* dependence is needed.

## High-grade (WHO 5th edition)/large B-cell lymphoma (ICC) with 11q aberrations (ICC 2022) (HG/LBCL-11q)

Burkitt-like lymphoma with 11q aberration was included in the WHO-HAEM4R as a provisional entity [41] to describe lymphomas that resemble BL morphologically and immunophenotypically, but lack *MYC-*R and carry typical chromosome 11q alterations; proximal gains (with a minimal region of gain in 11q23.2–23.3); and telomeric losses of 11q24.1-ter [56]. They usually have a nodal presentation with a median age at diagnosis of 15.5 years (range 4–52) [41, 71, 72]. Cytomorphology is reminiscent of BL as well as high-grade B-cell lymphomas and conspicuous coarse apoptotic debris in starry sky macrophages is typically seen [56, 73]. In contrast to BL, these cases show LMO2 and CD56 positivity, and are usually MUM1, EBER, and MYC negative [56, 68]. The entity has been updated to high-grade B-cell lymphoma with 11q aberrations in the WHO 5th edition and large B-cell lymphoma with 11q aberrations in the ICC 2022. For simplicity and although this session occurred prior to the classification changes, we will used the term HG/LBCL-11q.

HG/LBCL-11q have a distinct molecular profile different from *MYC-*positive BL having a more complex genetic aberration background than BL (gains in chromosome 5q, 12p, and 18q as well as deletions in 6q) [56, 68–70]. They also show a different mutational background with recurrent mutations in *GNA13* among other genes; BL-associated mutations of *TCF3* and *ID3* genes are absent in BLL 11q [68, 70].

We received four cases with the proposed diagnosis of HG/LBCL-11q (LYWS-333, 377, 589 and 648 cases). The HG/LBCL-11q cases received, comprised 1 female, 1 male and in two cases sex of the patient was not provided. Age at diagnosis ranged from 12 to 79 years old (median of 37 years). Although it is predominantly a lymphoma of children and young adults, rare cases in the elderly have also been reported [74, 75]. Two cases involved the nasopharynx, one the small intestine and another one soft tissue. One 79-year-old patient might be considered to have an age-related immunosuppression (LYWS-377, Rymkiewicz G., Maria Sklodowska-Curie National Institute of Oncology, Warsaw, Poland) while another suffered from Crohn’s disease (LYWS-333, Masaoutis Ch., Evangelismo General Hospital of Athens; Athens; Greece). Both cases were EBER-negative. Two patients received immunochemotherapy and achieved complete response, while clinical course of the other two cases was not known. Three out of the four cases showed high-grade morphology and one a classic Burkitt appearance. The infiltrate was diffuse in three cases and mixed with both nodular and diffuse pattern in one. In all cases a marked starry sky pattern could be seen. In LYWS-333 different morphologic areas ranging from blastoid to Burkitt-like were identified. The neoplastic cells expressed CD20, CD10, and BCL6 in all cases and were negative for TdT, EBER, and BCL2 in all cases as well. MYC expression was lower than 40% of neoplastic cells in 3 out of the 4 cases. Ki67 was high in all cases, 95–100% of neoplastic cells. *MYC*-R was not found in any case, although LYWS-377 case showed three copies of the gene. No rearrangements of either *BCL2* or *BCL6* were found in any case. Karyotype was complex in 3 out of the 4 cases (using conventional cytogenetics, high-resolution SNP array, array genomic hybridization, and/or triple color probe FISH studies). One of the cases (LYWS-648 case; Chen M, Pathology Department; UTSW Medical Center; Dallas; USA) was studied using NGS and mutations in *EZH2*, *KMT2D*, and *ERCC2* genes were identified.

**Fig. 6:**
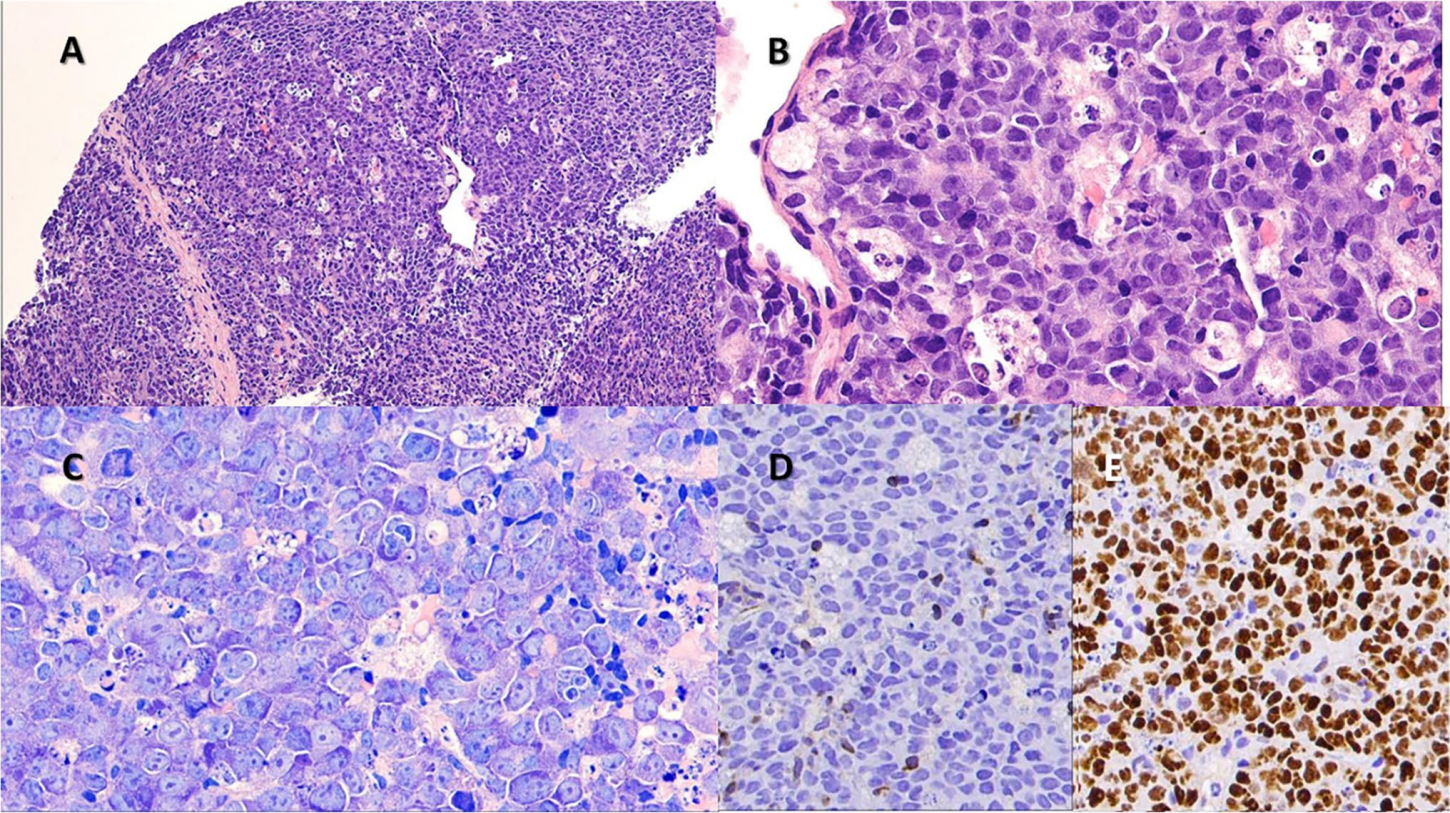
Aggressive B-cell lymphoma with MYC-R and 11q aberration (case 694). This case was problematic in classification. It had high-grade features with starry sky and tingible body macrophages with coarse apoptotic debris (**A**, H&E, 10 ×) seen in HG/LBCL-11q. However, the cytomorphology on H&E stain (**B**, 40 ×) and Giemsa stain (**C**, 50 ×) is closer to diffuse large B-cell lymphoma. The cells lacked BCL2 (**D**, 40 ×) but highly expressed MYC (**E**) by immunostaining. This case had both *MYC*-R and the 11q aberration

There were two cases with both 11q gain/loss aberrations and *MYC*–R (LYWS-694 and 510). LYWS-510 was noted above in the BL section, as the final decision was to consider this as BL. LYWS-694 (Húll KS; Pathology Department; Robert-Bosch-Krankenhaus, Stüttgart, Germany) presented as systemic disease involving small bowel and lymph nodes in a 41-year-old man. The patient suffered from immunodeficiency (lymphomatoid granulomatosis in 2007 and common variable immune deficiency in 2019). Clinical follow-up or treatment regimens were not known. Cytologically, LYWS-694 more closely resembled diffuse large B-cell lymphoma (Fig. 6). Neoplastic cells expressed CD20, CD10, MYC, and BCL6 and were negative for EBER, BCL2, and TDT. Ki67 was positive in nearly 95% of neoplastic cells.

LYWS-694 case highlights that fact the 11q abnormalities can be seen in a wide variety of morphologic contexts, but exact classification was uncertain and the submitted descriptive diagnosis of aggressive B-cell lymphoma with *MYC*-*R* and 11q aberration was accepted. The 11q gain/loss is not a distinctive feature for HG/LBCL-11q since it can also occur in *MYC*-positive BL and *MYC*-positive HGBL-nos, HGBLs with either DT/TH and in up to 16% of transformed FL [75, 76]. This suggests that 11q aberrations could be either a primary or a secondary genetic change in the development of aggressive B-NHLs. Indeed, 11q alterations can take place in BL progression [77, 78]. Thus, it is worth emphasizing that the 11q aberration in the context of a case with appropriate histomorphology and lack of *MYC*-R helps define this entity. Moreover, there is a need for refinement of minimal cytogenetic criteria and allowable morphologic variation for this entity since some cases may not resemble BL. Recent studies suggest that some cases more closely resembling the morphology of DLBCL may be acceptable in this entity [70].

## Pleomorphic/blastoid mantle cell lymphoma

We received 7 samples that could be considered as pleomorphic/blastoid mantle cell lymphoma (P/B-MCL) cases from 6 patients. This is an aggressive form of MCL characterized by pleomorphic large cell or blastoid morphology. The cases received highlighted the fact that aberrant phenotypic features such as CD10 and BCL6 expression may occur in P/B-MCL and raised issues in the diagnosis of cyclin D1-negative P/B-MCL. All cases were re-evaluated by the panel and 3 of 6 patients were considered as having P/B-MCL (LYWS**-**144 a and b, 449, and 785 cases).

For LYWS-144 case (Magno C et al. University of Pennsylvania; Philadelphia; USA), a 60-year-old man with diffuse lymphadenopathy and renal mass, two samples at different time points of the disease (diagnosis and recurrence four months later) were received. At diagnosis, the neoplastic cells showed a classic morphology and expressed CD20, CD5, cyclin D1, BCL2, BCL6, MUM1, and low proliferation index (25%). No molecular studies were done. The patient received high-intensity chemotherapeutic regimens suffering recurrence 4 months later. At recurrence, the cells were blastoid, overexpressed MYC, and had a higher proliferation index (75%). BCL6 and MUM1 were expressed in a subset of cells (30%). Interestingly, the relapse sample showed *MYC*, *BCL6* rearrangements on top of a *CCND1* gene rearrangement. The differential diagnosis with a HGBCL with *MYC* and *BCL6* rearrangement with *CCND1* rearrangement as a late event (third hit) would have been difficult to resolve without the biopsy specimen at initial presentation which already was cyclin D1-positive [61]. Case LYWS-449 (Bonometti A et al. University of Pavia; Pavia; Italy) was from a 45-year-old man with bone marrow and soft tissue involvement by lymphoma. A muscle biopsy was infiltrated by large pleomorphic cells expressing CD5, CD20, cyclin D1, BCL6, MUM1, MYC (> 80%), P53, and Ki67 (> 95%) and harbored *CCND1* and *MYC* rearrangements by FISH. Case LYWS-785 was a nasal mass from a 69-year-old woman with intermediate to large cells and a starry sky pattern. It expressed CD10, CD20, BCL6, MUM1, cyclin D1, and MYC (70%) but lacked CD5. By FISH, *CCND1* was rearranged and while extra copies of *MYC* were present, rearrangement could not be confirmed. This CD10 + phenotype fits with a prior report of CD10 + mantle cell lymphomas showing strong association with blastoid/pleomorphic morphology [79].

MCL patients with *MYC*-R are reported to have more often blastoid/pleomorphic morphology; a higher frequency of CD10, MYC, and BCL2 expression; a higher Ki67 proliferation rate; and poor outcome [80]. *MYC*-R may be present at initial diagnosis or develop during the course of the disease [81]. MCL with *BCL6*-R are exceptional cases and if other features associated with MCL are not present, one may consider DLBCL as an alternate diagnosis [82–84]. Exceptional cases with quadruple-hit rearrangement have also been reported [85]. Aberrant phenotypes have been described in MCL cases, mostly in association with blastoid/pleomorphic variants, including absence of CD5 and expression of LEF1, CD10, and BCL6. This further highlights the overlapping features between entities in progressed/transformed settings that preclude unequivocal classification [79, 86–88].

The two cases (LYWS-258 and 687) without *CCND1* rearrangement lacked cyclin D1 expression but were CD20, CD5, and SOX11 positive, suggesting that these may represent cyclin D1-negative blastoid/pleomorphic MCL [89]. Both presented with skin masses. Skin involvement in MCL is rare in general (1% of MCL cases) and has a high tendency to involve the legs (58% of cases). Blastoid morphology appears to be much more frequent than classical at this site (86% of cases in one series) [90]. Cutaneous MCL cases may have an unusual phenotype and are usually BCL2, MUM1, IgM, CD10, and BCL6 positive. This may make distinguishing cutaneous P/B-MCL from DLBC-leg-type or follicular lymphoma (primary cutaneous or systemic with secondary skin involvement) with a diffuse pattern challenging, unless cyclin D1 is also evaluated [90, 91]. MCL cases lacking *CCND1* rearrangement show *CCND2/CCND3* overexpression/rearrangement or *CCNE1/2* overexpression/*CDKN2A* homozygous deletions [16]. None of these features was present in these submitted two cases (studies performed in the laboratory of Dr. Elias Campo); the panel lacked molecular evidence to support a diagnosis of cyclin D1-negative MCL with P/B morphology.

Case LYWS-258 (Garamvölgyi E; et al.: University Hospital; Basel, Switzerland) was from an 89-year-old man with skin and retroperitoneal masses containing immunoblastic cells expressing CD20, CD5, SOX11, MYC, and Ki67 (90%). The cells had *MYC* and *BCL6* rearrangements but lacked *CCND1* rearrangement. It was felt to be best considered, as the submitters did, a high-grade B-cell lymphoma with *MYC* and *BCL6* rearrangement with CD5 and SOX11 expression. Case LYWS-687 (Kinney MC, et al.; Pathology and Laboratory Medicine; UTHSCSA; San Antonio; USA) was problematic since the material presented was from widely disseminated relapsed disease with a prior primary cutaneous leg type DLBCL. The relapsed material showed CD5 and SOX11 expression as well as *MYC-*R but no *CCND1*, *BCL2*, or *BCL6* rearrangement. Since the primary material was not reviewed and the molecular features of cyclin D1-negative MCL were not present, the panel considered this as compatible with relapsed primary cutaneous DLBCL, leg type with an unusual phenotype.

The final case, LYWS-762 (Parrott AM and colleagues, Columbia University, New York), has been already published as a case report as a primary DLBCL of the CNS in an 81-year-old man with both cyclin D1 expression and *CCND1*-R [92]*.* The large neoplastic cells expressed CD20 but lacked CD5, SOX11, and CD10. *BCL6*-R was present but no *BCL2* or *MYC*-*R* or increased signals for *BCL2*, *MYC*, and *IGH* were present. Two similar cases are present in the literature [93, 94]. It is reported that cyclin D1 could be overexpressed in a subgroup of DLBCL cases without CD5 or SOX11 expression and without *CCND1*-R [95]*.* In most of these cases either *BCL6* and/or *MYC* are translocated [95]. Whether the *CCND1*-R in this case could be secondary is also a consideration [61]. Studies for abnormalities seen in primary CNS DLBCL such as *MYD88* or *CD79B* mutations or MCL-associated mutations were not done in this case. We recognize that different observers might have an alternate interpretation of such a case and the panel felt this case was difficult to classify and as such agreed with the submitted somewhat descriptive diagnosis of primary DLBCL of the CNS with cyclin D1 expression and *CCND1*-R.

The P/B-MCL cases submitted illustrated the propensity to vary from the classic CD5 + /CD10-/BCL6- phenotype. SOX11 expression can help recognize cyclin D1-negative MCL. Even with use of the most appropriate and specific primary antibody, SOX11 can be expressed by other small B-cell lymphomas, some DLBCL, BL, and LBL [15]. In the absence of classic MCL morphologic and phenotypic features, demonstrating characteristic molecular features in other cyclin-family genes that have been identified is advisable to make a confident diagnosis of cyclin D1-negative classic MCL or P/B-MCL.

## Summary

Session 4 covered high-grade B-cell lymphomas and other uncommon diffuse aggressive B-cell lymphomas recognized by molecular features that may not be entirely specific. The overlapping features between various entities and resulting diagnostic challenges were highlighted by the submitted cases and emphasize the need for detailed immunophenotypic and molecular genetic characterization. As our experience with these cases grows, terminology is evolving to reflect our understanding of these entities and allowable morphologic and molecular genetic features. The experiences in this workshop informed the updates in disease classification for both the WHO 5th edition and 2022 International Consensus Conference publications.

## Take home messages

- DHL/THLs are aggressive lymphomas with differing morphologies (DLBCL-like, HGBCL-like, BL-like, or blastoid) that can occur de novo or as a transformation from prior lymphomas.
- Only cases with *MYC* and *BCL2* and/or *BCL6* gene rearrangements (as opposed to copy number abnormalities) should be included in DH/THL category.
- DHLs with *MYC/BCL2-*R and *MYC/BCL6*-R should be segregated.
- Commercially available *MYC* break apart FISH probes do not identify all *MYC*-R cases and addition of *IGH::MYC* dual fusion FISH studies will increase one’s ability to identify DHLs.
- TdT expression can be seen in DHL/THL with different morphologies but showing a common molecular background resembling FL. These should be diagnosed as DHL/THL with TdT expression rather than lymphoblastic lymphoma.
- Rare de novo B-LBL with *MYC*-R or DH genetics may occur in which *MYC-*R occurs during VDJ recombination.
- Large B-cell lymphoma with *IRF4*-R is present in children and adults, usually in the head and neck region or in gastrointestinal tract. They show a characteristic mutational profile with frequent *CARD11* and *IRF4* gene mutations. The identification of these cases, especially in younger patients, is important due to their favorable outcome. *IRF4*-R testing may be appropriate in younger patients with follicular grade 3B and DLBCL morphology coexpressing BCL6 and strong MUM1.
- Cases resembling BL morphologically, but lacking MYC expression, showing a conspicuous coarse apoptotic debris in starry sky macrophages or nodal presentation should be investigated for 11q aberrations.
- SOX11 is also highly characteristic of mantle cell lymphoma, but not specific. Suspected cases of P/B cyclin D1-negative MCL expressing SOX11 should ideally be investigated for *CCND2/3* expression/rearrangement for confident diagnosis.
- P/B-MCL could show either *MYC* and/or *BCL6* rearrangements, usually a secondary event; these cases show peculiar immunophenotype.

### Supplementary information

Below is the link to the electronic supplementary material.

Supplementary file1 (DOC 32 KB)

Supplementary file2 (XLSX 16 KB)

Supplementary file3 (DOCX 13 KB)

Supplementary file4 (XLS 64 KB)
