# Correction to: Mediastinal large B cell lymphoma and surrounding gray areas: a report of the lymphoma workshop of the 20th meeting of the European Association for Haematopathology

**DOI:** 10.1007/s00428-023-03678-4

**Published:** 2023-10-13

**Authors:** Sarah E. Gibson, Stefan Dojcinov, Snjezana Dotlic, Sylvia Hartmann, Eric D. Hsi, Monika Klimkowska, Federica Melle, Stefano A. Pileri, Colleen A. Ramsower, Karen Rech, Lisa M. Rimsza, Socorro Maria Rodriguez‑Pinilla, Thomas A. Tousseyn, Daphne de Jong, Elena Sabattini

**Affiliations:** 1 Division of Hematopathology, Department of Laboratory Medicine and Pathology, Mayo Clinic, Phoenix, AZ USA; 2 Department of Pathology, Morriston Hospital, Swansea Bay University Health Board, Swansea, UK; 3 Department of Pathology and Cytology, University Hospital Centre Zagreb, Zagreb, Croatia; 4 Dr. Senckenberg Institute of Pathology, Goethe University Frankfurt Am Main, Frankfurt Am Main, Germany; 5 Department of Pathology, Wake Forest University School of Medicine, Winston-Salem, NC USA; 6 Department of Clinical Pathology and Cancer Diagnostics, Karolinska University Hospital, Stockholm, Sweden; 7 Division of Haematopathology, IEO, European Institute of Oncology IRCCS, Milan, Italy; 8 Department of Research, Mayo Clinic, Scottsdale, AZ USA; 9 Department of Laboratory Medicine and Pathology, Mayo Clinic, Rochester, MN USA; 10 Pathology Department, Hospital Universitario Fundación Jiménez Díaz, Madrid, Spain; 11 Department of Imaging and Pathology, Translational Cell and Tissue Research Lab, KU Leuven, Louvain, Belgium; 12 Department of Pathology, Amsterdam UMC, Location VUMC, De Boelelaan 1117, 1081HV Amsterdam, The Netherlands; 13 Haematopathology Unit, IRCCS Azienda Ospedaliero-Universitaria Di Bologna, Bologna, Italy

**Correction to: Virchows Archiv**

10.1007/s00428-023-03550-5

In this article the author’s name "Stefan Dojcinov" was incorrectly written as "Stefan Docjinov".

The original article has been corrected.

