# Correction to: Aggressive B-cell non-Hodgkin lymphomas: a report of the lymphoma workshop of the 20th meeting of the European Association for Haematopathology

**DOI:** 10.1007/s00428-023-03677-5

**Published:** 2023-10-13

**Authors:** Socorro Maria Rodriguez-Pinilla, Stefan Dojcinov, Snjezana Dotlic, Sarah E. Gibson, Sylvia Hartmann, Monika Klimkowska, Elena Sabattini, Thomas A. Tousseyn, Daphne de Jong, Eric. D. Hsi

**Affiliations:** 1 Pathology Department, Hospital Universitario Fundación Jiménez Díaz, Madrid, Spain; 2 Department of Pathology, Morriston Hospital, Swansea Bay University Health Board, Swansea, UK; 3 Department of Pathology and Cytology, University Hospital Centre Zagreb, Zagreb, Croatia; 4 Division of Hematopathology, Department of Laboratory Medicine and Pathology, Mayo Clinic, Phoenix, AZ USA; 5 Dr. Senckenberg Institute of Pathology, Goethe University Frankfurt Am Main, Frankfurt Am Main, Germany; 6 Department of Clinical Pathology and Cancer Diagnostics, Karolinska University Hospital, Stockholm, Sweden; 7 Haematopathology Unit, IRCCS Azienda Ospedaliero-Universitaria Di Bologna, Bologna, Italy; 8 Department of Imaging and Pathology, Translational Cell and Tissue Research Lab, KU Leuven, Leuven, Belgium; 9 Department of Pathology, Amsterdam UMC, Location VUMC, De Boelelaan 1117, 1081HV Amsterdam, The Netherlands; 10 Department of Pathology, Wake Forest University School of Medicine, Winston-Salem, NC USA

**Correction to: Virchows Archiv**

10.1007/s00428-023-03579-6

In this article the author’s name Stefan Dojcinov was incorrectly written as Stefan Docjinov.

The original article has been corrected.

