# Supplementary material for: Aggressive B-cell non-Hodgkin lymphomas: a report of the lymphoma workshop of the 20th meeting of the European Association for Haematopathology
Source: Virchows Arch. 2023 Aug 2;484(1):15–29. doi: 10.1007/s00428-023-03579-6 (PMC10791773; doi:10.1007/s00428-023-03579-6)
Supplement: Supplementary file 1 — Supplementary file1 (DOC 32 KB) [file 428_2023_3579_MOESM1_ESM.doc]

**SUPPLEMENTARY MATERIAL AND METHODS.**

**DNA extraction.**

For mutation study, we extracted genomic DNA (DNAg) of tumoral FFPE samples using a cobas® DNA Sample Preparation Kit (Roche) in accordance with the manufacturer’s protocol. DNAs were quantified with Qubit® (Invitrogen, Carlsbad, CA, USA). The quality of the DNAs was studied with DNA Screen Tape kit and Tape Station technology (Agilent Technologies, Santa Clara, CA, USA).

**Mutation with SOPHiA DDM™ Lymphoma Solution.**

The SOPHiA DDM™ Lymphoma Solution Y05 (REF: B1.H1.0016.R-16, Sophia Genetics SA, Saint Sulpice, Switzerland) consisted of 54 genes. The target genes are listed in Supplementary table 1. The DNA libraries were performed according to manufacturer’s instructions (Sophia Genetics SA, Saint Sulpice, Switzerland). DNA libraries were generated from 200ng of DNA using the SOPHiA DNA Library Preparation Kit II (REF: 900232) and SOPHiA DDM™ Lymphoma Solution Y05 according to the manufacturer’s instructions (Sophia Genetics SA, Saint Sulpice, Switzerland). After library preparation, indexing and bead purification, the libraries were quantified by Qubit® (Invitrogen, Carlsbad, CA, USA) and then pooled and normalized for sequencing. The pooled libraries were sequenced with a Miseq Reagent Kit V3 (paired-end, 2x300) on a MiSeq instrument (Illumina, San Diego, CA, USA), as described in the manufacturer’s protocol. The analysis was performed using the [SOPHiA DDM™ platform](https://www.sophiagenetics.com/technology) (Sophia Genetics SA, Saint Sulpice, Switzerland).
