# Supplementary material for: Aggressive B-cell non-Hodgkin lymphomas: a report of the lymphoma workshop of the 20th meeting of the European Association for Haematopathology
Source: Virchows Arch. 2023 Aug 2;484(1):15–29. doi: 10.1007/s00428-023-03579-6 (PMC10791773; doi:10.1007/s00428-023-03579-6)
Supplement: Supplementary file 3 — Supplementary file3 (DOCX 13 KB) [file 428_2023_3579_MOESM3_ESM.docx]

**Supplementary Table 2. Target Genes Lymphoma Panel.** The SOPHiA DDM™ Lymphoma Solution included 54 genes implicated in Lymphomas (Sophia Genetics).

| ARID1A | FOXO1 | NFKBIE | ATM | FBXW7 | XPO1 |
| --- | --- | --- | --- | --- | --- |
| B2M | GNA13 | PAX5 | BCL6 | KRAS |  |
| BCL2 | ID3 | PIM1 | BRAF | NOTCH1 |  |
| CCND3 | IRF4 | PRDM1 | BTK | NOTCH2 |  |
| CD58 | MAL | PTPN1 | CARD11 | NRAS |  |
| CDKN2A | MEF2B | REL | CCND1 | PLCG2 |  |
| CDKN2B | MLL | SOCS1 | CD79A | PTEN |  |
| CIITA | MYC | TNFAIP3 | CD79B | SF3B1 |  |
| CXCR4 | MLL2 | TNFRSF14 | CREBBP | STAT6 |  |
| EP300 | MYD88 | TP53 | EZH2 | TCF3 |  |
